# Fatal Effect from Vaccination

**Published:** 1829

**Authors:** 


44. *Fatal effect from Vaccination.*—Having heard that a citizen of Cincinnati fell a victim to the vaccine disease in the month of January, we requested of Dr. J. K. Sparks, one of the vaccinators of the poor, to furnish us with some account of the case, and are enabled to lay before the profession the following statement, extracted from his letter:

"On the 21st January, 1829, I vaccinated Mrs. Fisher and her three children. The night of the 26th of the same month Nymphis Fisher, her husband, *who had been vaccinated and had the disease regularly many years before*, took matter from his little daughter's arm, and with the point of a pin vaccinated himself. Within two or three hours the place began to itch and pain him; he was chilly and had a restless night.

Tuesday, 27th, he attempted to gather ashes with his cart (for that was his daily business) but before noon he was so unwell that he returned home and took to his bed. His wife poulticed his arm and gave him a dose of medicine, but he still got worse.

Wednesday, 28th, in the afternoon I was sent for, but it was night before I saw him. His tongue had a thick bilious coating; his skin and eyes were yellow; his pulse frequent, and he was very restless. On the left arm above the elbow, where he was vaccinated, there was a tumour as large as an ordinary hen's egg, and almost as abrupt in its figure as if one had heen inserted into the cellular membrane under the skin. I first gave him a purge of calomel and sulphate of magnesia; then small doses of calomel and Dover's powder, with a subsequent purge of compound powder of jalap.

Thursday, 29th, I applied the roller bandage, beginning at the ends of the fingers, and continuing it to the arm-pit; which arrested the swelling for a time. The alterative and cathartic medicines were continued.

Friday, 30th, a thin, yellow, fœtid matter began to discharge from about the tumour. The above treatment continued.

Saturday, 31st, the fœtid matter was more copious, and the muscles of the arm were flaccid, and would hang down when the bandage was off. The same prescription continued.

Sunday afternoon, 1st February, the whole arm greatly swelled, I applied blistering plasters, which covered the arm generally, from the shoulder to the wrist, and put the bandage pretty tight over them. Sulph. quin. and Port wine were ordered.

Monday, 2d, the plasters had acted well, and where there was but little pressure the blisters were fiilled with yellow matter. The arm was dressed with a mild ointment. Brandy and bark were given freely. Dr. Slayback was called in—recommended a weak ley poultice to extend from the shoulder to the wrist.

Tuesday, 3d, a stronger ley poultice was ordered, which irritated the arm so much, that he refused to let it stay on. A yeast poultice was then applied to the whole arm. Gangrene had taken place above the elbow, and it was much swelled to the hand.

Wednesday, 4th, the bark and brandy were continued and the yeast poultice reapplied. But as the vital powers gave way, the gangrene and mortification extended over the whole limb. In the latter part of the night he died.

My opinion is, that if Mr. F. had not been vaccinated he would have had an attack of illness, which might have proved fatal as he was a weakly man; but the vaccine matter, (though genuine as was proved by its acting favourably on his little daughter) was poisonous to his disordered system."

May, 1829.

